# Supplementary material for: Incidence and predictors of mortality among neonates with respiratory distress syndrome admitted at West Oromia Referral Hospitals, Ethiopia, 2022. Multi-centred institution based retrospective follow-up study
Source: PLoS One. 2023 Aug 1;18(8):e0289050. doi: 10.1371/journal.pone.0289050 (PMC10393157; doi:10.1371/journal.pone.0289050)
Supplement: S1 Checklist — (DOCX) [file pone.0289050.s001.docx]

STROBE Statement—checklist of items that should be included in reports of observational studies

|  | Item No. | Recommendation | Page  No. | Relevant text from manuscript |
| --- | --- | --- | --- | --- |
| **Title and abstract** | 1 | (*a*) Indicate the study’s design with a commonly used term in the title or the abstract | 1 | Retrospective Follow-up Study. |
|  |  | (*b*) Provide in the abstract an informative and balanced summary of what was done and what was found | 2 | A retrospective follow-up study was conducted among 406 neonates admitted with respiratory distress syndrome at five referral hospitals from January 1, 2019 to December 31, 2021 in West Oromia, Ethiopia. A simple random sampling technique was employed. Data were collected using a structured checklist. The data entered in to Epi data version 4.6.0.2 and exported to STATA Version 14 for cleaning, coding and analysis. The Kaplan–Meier curve was used to estimate survival time. The Weibull regression model was fitted to identify the predictors of mortality and variables with a P-value < 0.05 was taken as significant predictors of mortality.  Result: A total of 406 neonates with respiratory distress syndrome medical records participated in the study. The overall incidence of neonatal mortality was 59.87/1000 neonates-days observations (95%CI: 51.1-70.2). The proportion of death was 152 (37.44%) (95% CI: 32.7-42.2). The median survival time of follow-up was 11 days (95% CI: 10-23). Very low birthweight (AHR=4.5, 95%CI: 2.0-10.9) and low birth weight (AHR=3.1, 95%CI: 1.4-6.6), perinatal asphyxia (AHR= 2.7, 95%CI: 1.8-4), Chorioaminoitis (AHR=2.2, 95%CI: 1.4-3.5) and multiple pregnancies (AHR= 2.2, 95%CI: 1.4-3.4) increased the hazard of death, whereas, antenatal corticosteroid administration (AHR=0.33, 95%CI: 0.2-0.7) was negatively associated with neonatal mortality. |
| Introduction | | | |  |
| Background/rationale | 2 | Explain the scientific background and rationale for the investigation being reported | 3, 4 |  |
| Objectives | 3 | State specific objectives, including any prespecified hypotheses | 2 | To determine the incidence of mortality among neonates admitted with respiratory distress syndrome in West Oromia referral hospitals, Ethiopia, 2022.  To identify the predictors of mortality among neonates admitted with respiratory distress syndrome in West Oromia Referral hospitals, Ethiopia, 2022. |
| Methods | | | |  |
| Study design | 4 | Present key elements of study design early in the paper | 5 |  |
| Setting | 5 | Describe the setting, locations, and relevant dates, including periods of recruitment, exposure, follow-up, and data collection | 5, 6, and 8 |  |
| Participants | 6 | (*a*) *Cohort study*—Give the eligibility criteria, and the sources and methods of selection of participants. Describe methods of follow-up  *Case-control study*—Give the eligibility criteria, and the sources and methods of case ascertainment and control selection. Give the rationale for the choice of cases and controls  *Cross-sectional study*—Give the eligibility criteria, and the sources and methods of selection of participants | 5, 8 |  |
|  |  | (*b*) *Cohort study*—For matched studies, give matching criteria and number of exposed and unexposed  *Case-control study*—For matched studies, give matching criteria and the number of controls per case |  |  |
| Variables | 7 | Clearly define all outcomes, exposures, predictors, potential confounders, and effect modifiers. Give diagnostic criteria, if applicable | 7, 8 | **Neonatal Respiratory Distress Syndrome:** diagnosed based on the presence of one or more of the following signs: an abnormal respiratory rate, expiratory grunting, nasal flaring, and chest wall recessions with or without cyanosis.  **Event (death**):- Neonate died in the hospital who was admitted with RDS. |
| Data sources/ measurement | 8* | For each variable of interest, give sources of data and details of methods of assessment (measurement). Describe comparability of assessment methods if there is more than one group | 7, 8 |  |
| Bias | 9 | Describe any efforts to address potential sources of bias |  |  |
| Study size | 10 | Explain how the study size was arrived at | 6 |  |

Continued on next page

| Quantitative variables | 11 | Explain how quantitative variables were handled in the analyses. If applicable, describe which groupings were chosen and why |  |  |
| --- | --- | --- | --- | --- |
| Statistical methods | 12 | (*a*) Describe all statistical methods, including those used to control for confounding | 9 |  |
|  |  | (*b*) Describe any methods used to examine subgroups and interactions |  |  |
|  |  | (*c*) Explain how missing data were addressed |  |  |
|  |  | (*d*) *Cohort study*—If applicable, explain how loss to follow-up was addressed  *Case-control study*—If applicable, explain how matching of cases and controls was addressed  *Cross-sectional study*—If applicable, describe analytical methods taking account of sampling strategy | 7 | **Censored**: new-borns with RDS who did not develop the outcome of interest (death) until the end of the follow-up period or recovery from illness, or discharged against medical advice. |
|  |  | (*e*) Describe any sensitivity analyses |  |  |
| Results | | | | |
| Participants | 13* | (a) Report numbers of individuals at each stage of study—eg numbers potentially eligible, examined for eligibility, confirmed eligible, included in the study, completing follow-up, and analysed |  |  |
|  |  | (b) Give reasons for non-participation at each stage |  |  |
|  |  | (c) Consider use of a flow diagram |  |  |
| Descriptive data | 14* | (a) Give characteristics of study participants (eg demographic, clinical, social) and information on exposures and potential confounders | 9-15 |  |
|  |  | (b) Indicate number of participants with missing data for each variable of interest |  |  |
|  |  | (c) *Cohort study*—Summarise follow-up time (eg, average and total amount) | 15, 16 |  |
| Outcome data | 15* | *Cohort study*—Report numbers of outcome events or summary measures over time | 15 | From the follow-up outcome, 3.45% were lost to follow-up or left the hospitals without health professionals' permission or against medical advice while 37.4% were died |
|  |  | *Case-control study—*Report numbers in each exposure category, or summary measures of exposure |  |  |
|  |  | *Cross-sectional study—*Report numbers of outcome events or summary measures |  |  |
| Main results | 16 | (*a*) Give unadjusted estimates and, if applicable, confounder-adjusted estimates and their precision (eg, 95% confidence interval). Make clear which confounders were adjusted for and why they were included |  |  |
|  |  | (*b*) Report category boundaries when continuous variables were categorized |  |  |
|  |  | (*c*) If relevant, consider translating estimates of relative risk into absolute risk for a meaningful time period |  |  |

Continued on next page

| Other analyses | 17 | Report other analyses done—eg analyses of subgroups and interactions, and sensitivity analyses |  |  |
| --- | --- | --- | --- | --- |
| Discussion | | | | |
| Key results | 18 | Summarise key results with reference to study objectives | 17, 18 |  |
| Limitations | 19 | Discuss limitations of the study, taking into account sources of potential bias or imprecision. Discuss both direction and magnitude of any potential bias | 25 |  |
| Interpretation | 20 | Give a cautious overall interpretation of results considering objectives, limitations, multiplicity of analyses, results from similar studies, and other relevant evidence | 18-25 |  |
| Generalisability | 21 | Discuss the generalisability (external validity) of the study results | 22-25 |  |
| Other information | |  | | |
| Funding | 22 | Give the source of funding and the role of the funders for the present study and, if applicable, for the original study on which the present article is based | 27 | Funding not applicable. |

*Give information separately for cases and controls in case-control studies and, if applicable, for exposed and unexposed groups in cohort and cross-sectional studies.

**Note:** An Explanation and Elaboration article discusses each checklist item and gives methodological background and published examples of transparent reporting. The STROBE checklist is best used in conjunction with this article (freely available on the Web sites of PLoS Medicine at http://www.plosmedicine.org/, Annals of Internal Medicine at http://www.annals.org/, and Epidemiology at http://www.epidem.com/). Information on the STROBE Initiative is available at www.strobe-statement.org.
