# Supplementary material for: Incidence and predictors of mortality among neonates with respiratory distress syndrome admitted at West Oromia Referral Hospitals, Ethiopia, 2022. Multi-centred institution based retrospective follow-up study
Source: PLoS One. 2023 Aug 1;18(8):e0289050. doi: 10.1371/journal.pone.0289050 (PMC10393157; doi:10.1371/journal.pone.0289050)
Supplement: Attachment — Submitted filename: Response to reviewers.docx [file pone.0289050.s003.docx]

**Response for Academic editor**

**We are thankful for your supportive and precious comments and suggestion.**

Journal Requirements

1. We have corrected it.

2. We re-stated all raised concerns.

3. We’ll uploaded the data set

4. We removed ethical related points and mentioned only in method section.

5. Corrected

**We thank you very much for your constructive comments, questions, suggestions and kind words.**

**Response for Reviewer1**

**Question:** The Cox regression model is widely used in medical research and the shape and scale parameters of baseline hazard are not taken into account. Since the proportionate hazard assumption was met (0.5725), the parametric survival regression analysis is not necessary.

**Answer:** Yeah, as you have mentioned Cox regression model is widely used in health researches. But we compared and selected the one with the lowest AIC to reduce the prediction error. This indicates Weibull regression predicts our data better than Cox regression model. Proportionate hazard assumption was met (0.5725), but it is not criteria to go to with Weibull or other parametric models. When the assumption failed to met, we go for extended Cox regression model not for parametric models. So when the proportional assumption failed, we can’t make analysis with parametric models rather we do the analysis with extended Cox regression models.

**Question:** Do you have any evidence that the bassline hazed distribution RDS existed before your study?

**Answer:** We don’t have but the Weibull regression model predicts/fits the data than other models which is indicative for less prediction error.

**Concern:** Your manuscript is not written in Standard English

**Answer:** We tried to improve the language by now.

**Question:** In the source population, section “all neonates’ charts”. Are going to conclude for records?

**Answer:** First, we thought that since it is a secondary data based study we mention as a chart but now we changed it to neonates.

**Question:** In the Sample size determination section, “(sample size was 384.16)”. Please clarify!

**Answer:** First, the sample size is 384.16 and after adding 10% considering missed data, the total sample size will be 422.576 which is equal to 423.

**Question:** Also, the first objective of your study was the incidence of mortality. Is that possible to calculate sample size by considering the single population formula?

**Answer:** Since there is no study conducted using hazard ratio as a method of measuring association, we used the first objective. In addition, we tried to use Relative Risk by converting Odds Ratio to Relative Risk then using RR as Hazard Ratio and at the last, it is less than the first objective’s sample size.

**Question:** In the study setting section, you do not mention the numbers of health workers in their respective professions –which would have a significant impact on your results and neonatal survival.

**Answer:** we corrected and added the raised concern.

**Question:** When using the term "PROM" you do not give the standard definition used in your study. Since the definition in use can have a relevant influence on the survival of the neonates you should clarify this issue.

**Answer:** We wrote the standard definition or we don’t get your concern clearly.

**Question:** In the data collection section, you stated, “supervised by the principal investigator”. Please clarify!

**Answer:** We corrected it. The supervision by the principal investigator was not data collection directly. The principal investigator was supervised the data at the last of the day of data collection.

**Question:** In data processing and analysis, you reported” Kaplan-Meier survival curve”, your title is the incidence of mortality, it is better to report Nelson Aalen cumulative hazard

**Answer:** corrected

**Question:** In the same section, you checked multicollinearity by a variance inflation factor. Please report the mean VIF or the cut point

**Answer:** we added it.

**Question:** How do you measure perinatal asphyxia 3.2% of the neonates were delivered at home?

**Answer:** The health professionals were asked for crying of the newborn at the time of birth and if the baby were not cried by then they report that as birth asphyxia.

**Question:** In your section “Incidence of mortality," you report the incidence of mortality in each hospital. In Nekemte Comprehensive Specialized Hospital 65.49 and 56.94 deaths / 1000neonates days in Mettu Karl Comprehensive Specialized Hospital. This disparity in the incidence of mortality needs a possible explanation and would greatly improve the relevance of your manuscript.

**Answer:** We added it.

**Question:** In the model comparison section, you reported both graphs and tables. Please report either table or graphs

**Answer:** Yes, We addressed in table using statistically while we used C-Snell chart graphically. If it is not necessary or better to remove one of the method we can do that.

**Question:** In the section on predictors of mortality among neonates with respiratory distress syndrome, you stated that “the test of equality for survival distribution for different categories of variables was performed with Kaplan-Meier and the log-rank test.” Please clarify!

**Answer:** Kaplan Meier curve (log-log plot) used graphically while long rank is statistically. Both of the method is used for checking proportionality assumption.

**Concern:** In Table 6, please add the percentage of cell value

**Answer:** Should we have to add percentage in the regression table?

**Response for Reviewer2**

**Concerns:** concerns regarding language and any points other than those mention under.

**Answer:** We tried to improve the concerns as per the given comments and suggestion**.**

**Question:** Line 120: Would you explain why you consider as exclusion criteria?

**Answer:** Because those neonates with major respiratory and cardiac system congenital malformations are highly prone for death, the case can hyper estimates the outcome of interest (death).

**Question:** Line 151-153: what is the value of operationalizing RDS? And also you already defined it on the introduction section.

**Answer:** We operationalize it because of how/when we say that the neonates diagnosed with RDS since RDS do have many differential diagnoses. So, the neonate must fulfil the above criteria to say the neonate has RDS.
