# Supplementary material for: Incidence and predictors of mortality among neonates with respiratory distress syndrome admitted at West Oromia Referral Hospitals, Ethiopia, 2022. Multi-centred institution based retrospective follow-up study
Source: PLoS One. 2023 Aug 1;18(8):e0289050. doi: 10.1371/journal.pone.0289050 (PMC10393157; doi:10.1371/journal.pone.0289050)
Supplement: Attachment — Submitted filename: Response to reviewers.docx [file pone.0289050.s004.docx]

**Ref:** PONE-D-22-27113R1

**Journal:** PLOS ONE

**Title:** Incidence and Predictors of Mortality among Neonates with Respiratory Distress Syndrome Admitted at West Oromia Referral Hospitals, Ethiopia, 2022. Multi-centred Institution Based Retrospective Follow-up Study.

**Editor:** Dereje Haile, MPH/RH

**Authors:** Bruck Tesfaye Legesse, Netsanet Melkamu Abera, Tewodros Getaneh Alemu, Kendalem Asmare Atalell

**Subject: Submission of revised manuscript**

**Dear Dereje Haile,**

Thank you for allowing us to revise our manuscript entitled “Incidence and Predictors of Mortality among Neonates with Respiratory Distress Syndrome Admitted at West Oromia Referral Hospitals, Ethiopia, 2022. Multi-centred Institution Based Retrospective Follow-up Study”. We are very pleased with the second reviewer’s and Editor’s comments. We have now carefully revised the manuscript and incorporated the comments. Our responses are given in point-by-point responses to each of the reviewers and the editor’s comments below. We have also attached the revised version of the manuscript with tracked changes and a clean version.

We hope the revised version is suitable for publication and look forward to hearing from you in due course.

Sincerely

Bruck Tesfaye Legesse

Corresponding Author

**Response for Academic editor**

We are thankful for your supportive and precious comments and suggestion. We have carefully revised the manuscript based on your comments.

Journal Requirements

1. Please review your reference list to ensure that it is complete and correct. If you have cited papers that have been retracted, please include the rationale for doing so in the manuscript text or remove these references and replace them with relevant current references. Any changes to the reference list should be mentioned in the rebuttal letter that accompanies your revised manuscript. If you need to cite a retracted article, indicate the article’s retracted status in the References list and also include a citation and full reference for the retraction notice.

**Author response:** Thank you so much for your insights, we have tried to revise the reference lists. Regarding retracted references we have tried to correct and replace them with the standard referencing system according to the journal requirements except for reference **number 03,** which is the source for the reference is a database based in the USA, a collection of 45 articles based on RDS and can’t be found on Google scholar, PubMed or other databases suitable for citation.

**Response for Reviewer1**

**Author response:** Thank you so much for your thoughtful comments and recommendations. We have revised the manuscript based on your comments.

1. Thank you for revising your manuscript in response to my comments.
2. **Author response:** Thank you dear reviewer for your appropriations for the previous revision. Please check the grammar. For example, in line #91, after "however," commas are needed! Please read the whole document.

**Author response:** We have revised the manuscript for language throughout the document including in line #91.

3. There is an inconsistency in the method section "1.1–1.4; please remove it.

**Author response:** Thank you so much, we have removed the above number and corrected the inconsistencies in the method sections.

4. You calculated the sample size using the conventional method, or 50%. However, a study conducted at Gondar Hospital reported that the probability of mortality among RDS children was 16.37. Hence, I recommend that you use the sample size method for the Cox model or the Freedman method by using the following Stata command:

Power cox, failprob (.1637) wdprob (0.05)

**Author response:** Thank you for your recommendation, but the article you mention has a different source population than our study which is conducted among neonates with Respiratory Distress (RD) which is an umbrella diagnosis for PNA, RDS, MAS, and other disorders cause’ respiratory distress. RDS is one parts of RD.
